# A randomised controlled trial comparing the effectiveness of tai chi alongside usual care with usual care alone on the postural balance of community-dwelling people with dementia: protocol for the TACIT trial (TAi ChI for people with demenTia)

**DOI:** 10.1186/s12877-018-0935-8

**Published:** 2018-11-03

**Authors:** Samuel R. Nyman, Christopher Hayward, Wendy Ingram, Peter Thomas, Sarah Thomas, Michael Vassallo, James Raftery, Helen Allen, Yolanda Barrado-Martín

**Affiliations:** 1 Department of Psychology and Ageing & Dementia Research Centre, Faculty of Science & Technology, Bournemouth University, Poole House, Talbot Campus, Poole, Dorset, BH12 5BB UK; 2 Peninsula Clinical Trials Unit, Peninsula Medical School, University of Plymouth, Drake Circus, Plymouth, Devon PL4 8AA UK; 3 Bournemouth University Clinical Research Unit, Faculty of Health and Social Sciences, Bournemouth University, Royal London House, Lansdowne Campus, Christchurch Road, Bournemouth, Dorset, BH1 3LT UK; 4 Centre of Postgraduate Medical Research and Education, Faculty of Health and Social Sciences, Bournemouth University, Royal London House, Lansdowne Campus, Christchurch Road, Bournemouth, Dorset, BH1 3LT UK; 5 Faculty of Medicine, University of Southampton, Building 85, Life Sciences Building, Highfield Campus, Southampton, SO17 1BJ UK

**Keywords:** Accidental falls, Behaviour change, Caregiver, Clinical trial, Dementia, Exercise, Feasibility, Postural balance, Tai chi

## Abstract

**Background:**

Falls are a public health issue for the older adult population and more so for people with dementia (PWD). Compared with their cognitively intact peers, PWD are at higher risk of falls and injurious falls. This randomised controlled trial aims to test the clinical and cost effectiveness of Tai Chi to improve postural balance among community-dwelling PWD and to assess the feasibility of conducting a larger definitive trial to reduce the incidence of falls among PWD.

**Methods:**

A 3-centre parallel group randomised controlled trial with embedded process evaluation. One hundred and fifty community-dwelling dyads of a person with dementia and their informal carer will be recruited and assessed at baseline and at six-month follow-up. Dyads will be randomised in a 1:1 ratio to either usual care or usual care plus a Tai Chi intervention for 20 weeks. The Tai Chi intervention will consist of weekly classes (45 min’ Tai Chi plus up to 45 min for informal discussion, with up to 10 dyads per class) and home-based exercises (20 min per day to be facilitated by the carer). Home practice of Tai Chi will be supported by the use of behaviour change techniques with the Tai Chi instructor at a home visit in week 3–4 of the intervention (action planning, coping planning, self-monitoring, and alarm clock reminder) and at the end of each class (feedback on home practice). The primary outcome is dynamic balance measured using the Timed Up and Go test, coinciding with the end of the 20-week intervention phase for participants in the Tai Chi arm. Secondary outcomes for PWD include functional balance, static balance, fear of falling, global cognitive functioning, visual-spatial cognitive functioning, quality of life, and falls. Secondary outcomes for carers include dynamic balance, static balance, quality of life, costs, and carer burden.

**Discussion:**

This trial is the first in the UK to test the effectiveness of Tai Chi to improve balance among PWD. The trial will inform a future study that will be the first in the world to use Tai Chi in a trial to prevent falls among PWD.

**Trial registration:**

NCT02864056.

## Background

Falls are the leading cause for emergency department (ED) presentation in adults aged 65 and above [1]. A significant proportion of these patients have dementia - around 25–34% [2, 3] - because people with dementia (PWD) are more than twice as likely to fall and twice as likely to experience injurious falls compared to their cognitively intact peers [4, 5]. The consequences are long-term and far-reaching; PWD are more likely to experience adverse health outcomes during their hospital stay and after discharge such as hospital readmission, institutionalisation, and mortality [6–8]. Thus, there is a pressing need to prevent falls among PWD.

There is robust evidence for interventions to prevent falls and fall-related injuries among community-dwelling people without dementia and, in particular, exercise-based interventions [9, 10]. However, research is required into the best ways to provide exercise-based interventions. For example, a recent large UK exercise trial found that home-based exercise did not increase activity or prevent falls [11]. For people with dementia, meta-analyses have found that strength and balance training interventions significantly prevent falls among older people with cognitive impairment in the community (pooled risk ratio = 0.68, 95% CI = 0.55–0.85) [12] and across settings (pooled rate ratio = 0.68, 95% CI = 0.51–0.91) [13]. However, few studies were included in the meta-analyses (3 community-dwelling; 7 across-settings) and some trials recruited small samples (as few as 11 participants in the intervention group). Further research is critical to determine which type of physical activity best prevents falls among PWD. Tai Chi may well be an effective way of promoting exercise for falls prevention [14]. Tai Chi is an ancient form of Chinese mind-body exercise, where participants carry out smooth and continuous body movements along with deep breathing and mental concentration [15]; equivalent to moderate-intensity exercise and quiet meditation [16]. This form of exercise is particularly suited for PWD with the use of slow and repetitive movements [17].

This protocol describes a randomised controlled trial (RCT) that aims to test the effectiveness of Tai Chi to improve postural balance among community-dwelling PWD and to assess the feasibility of conducting a definitive trial to reduce falls. We hypothesise that Tai Chi will help prevent falls by improving dynamic balance. Dynamic balance is highly predictive of rate of falls [18–20], and so it will be used as a surrogate outcome in this trial. This research programme is the first in the UK to test the effectiveness of Tai Chi to improve balance among PWD (current study) and the first in the world to use Tai Chi in a randomised controlled trial to prevent falls among PWD (future study). The trial is registered on ClinicalTrials.gov (ID no: NCT02864056, first posted August 11th, 2016) and this paper is a summary of the protocol version 4.3, dated 23/05/2018. To test if the new intervention can provide patient benefit in addition to usual care, no alternative intervention or waiting list control group will be offered.

## Methods/design

### Participants, interventions, and outcomes

#### Design

**Fig. 1:**
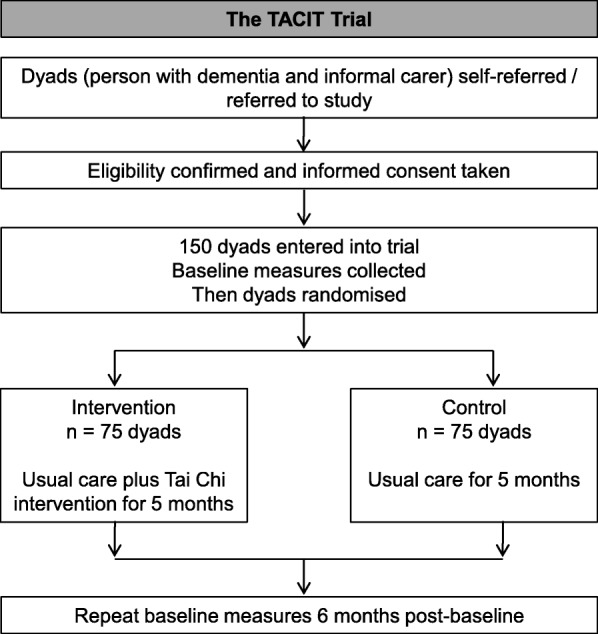
Flow chart of study participation

This is a randomised, assessor-blind, two-arm, parallel group, superiority trial, with an embedded intervention pilot study [21]. In this RCT, 150 dyads, each comprising a person with dementia and their informal carer, will be randomised to either the control group (usual care) or the intervention group (usual care plus the TACIT Tai Chi intervention) in a 1:1 ratio at each of the three treatment sites (see Fig. 1).

#### Study setting

This study will be conducted in three locations across the South of England (see ClinicalTrials.Gov registration for list of study sites). Participants will be identified and recruited via various sources, including National Health Service (NHS) research / clinic databases, memory assessment services (where patients are provided with a dementia diagnosis), local charities, and self-referral. Baseline and follow-up appointments will be conducted in participants’ homes or local venues. Tai Chi classes will be provided in suitable venues (e.g. church halls) and home-based Tai Chi instruction delivered in participants’ homes.

#### Study population

The target group for this study is people with mild to moderate dementia and their informal carer. Both the PWD and the carer must consent to participate in the study for the dyad to be eligible for inclusion. Informal carers may be the spouse, close relative, friend, or neighbour who live with the PWD participant or visit at least twice a week. While there can be more than one informal carer to support the PWD during the trial, data will be collected from only one primary informal carer.

#### Eligibility criteria for PWD

PWD must satisfy the following criteria to be enrolled in the study: Aged 18 or above, living at home, have a diagnosis of a dementia (indicated on their medical record held by the NHS or general practitioner [GP]), are physically able to do standing Tai Chi, and willing to attend weekly Tai Chi classes. The following exclusion criteria will be applied: Living in a care home; in receipt of palliative care; have severe dementia, a Lewy body dementia or dementia with Parkinson’s disease, or severe sensory impairment; are already currently practising or have been practising within the past 6 months Tai Chi or similar exercise (Qigong, yoga, or Pilates) on average once a week or more; are currently under the care of or have been referred to a falls clinic for assessment, or are currently attending a balance exercise programme (e.g. Otago classes); or lack mental capacity to provide informed consent. In regard to dementia severity, the Mini Addenbrooke’s Cognitive Examination (M-ACE) will be conducted at the initial visit after informed consent has been obtained [22]. Scores of 9 or less will be regarded as being indicative of severe dementia and grounds for exclusion from the trial. No ceiling cut-off will be used. In relation to types of dementia, the fall rates in those with either Lewy body dementia or dementia with Parkinson’s disease are significantly higher than other forms of dementias and so would require a different intervention (e.g. [23]).

#### Eligibility criteria for patient’s informal carers

Informal carers must satisfy the following criteria to be enrolled in the study: Able to commit to supporting the PWD by participating in data collection throughout the trial and in the intervention components, if allocated to the intervention group, physically able to do standing Tai Chi, and willing to attend weekly Tai Chi classes. The following exclusion criteria will be applied: Carers who have severe sensory impairment or lack mental capacity to provide informed consent.

#### Usual care

Both trial groups will continue to receive usual care during the course of the trial. In the UK, those who are concerned about their memory or are suspected to have dementia by their GP are referred to a memory assessment service. The memory assessment service will diagnose a person with dementia, and depending on the type and severity of dementia, may prescribe medication. Otherwise, no further treatments are offered for the PWD, and in particular, no Tai Chi or other physical activity is prescribed. Patients under the care of one of the NHS Trusts that are recruiting to the trial are provided with an educational course to provide information, support, and advice to PWD and a similar course is provided separately for informal carers. In addition, for those living in some areas, a referral is made to a memory advisor who provides telephone contact to check on the wellbeing of the PWD and their informal carer. Under the two other trusts, there is support from the voluntary sector in the form of memory advisors for every patient and informal carer before and after diagnosis, in the form of advice, information, guidance, and signposting to local services.

#### Control group: Usual care

Participants in the control group will be asked not to take up Tai Chi or similar exercise (Qigong, yoga, or Pilates) during the period of the project.

#### Intervention group: Tai chi in addition to usual care

The Tai Chi intervention comprises 3 components: (1) Tai Chi classes, (2) home-based Tai Chi exercises, and (3) a behaviour change component (see logic model in Table 1). The intervention has been designed for participants to accrue 50 h or more of Tai Chi physical activity, because exercise-based interventions to prevent falls are more effective if: they have a higher dosage (50 h or more) and challenge balance (e.g. exercises are conducted while standing) [24]. To allow sufficient time for participants in the intervention condition to be allocated to a class and to complete the 20-week intervention, the post-intervention outcome assessments are scheduled 6 months post-baseline, to coincide with the end of the 20-week intervention period. Information about the Tai Chi exercise prescription and instructional methods are provided in Table 2.

**Table 1:** Logic model of the TACIT Tai Chi intervention

Inputs	Activities	Outputs	Impacts	Outcomes
*Human resources:* • Tai Chi instructors to deliver the intervention • Research team to make telephone calls to remind dyads to attend classes *Products:* • Booklet to support practice of home-based Tai Chi • Homework sheets to support practice of home-based Tai Chi • Alarm clocks to help remind dyads to practise Tai Chi at home *Estates:* • Venues in the NHS / community accessible by public transport and that have free car parking for hire of the Tai Chi classes	*Intervention contact:* • Weekly 90 min Tai Chi class for 20 weeks (45mins Tai Chi, 45mins socialising/Q&A with instructor) • A home visit by Tai Chi instructor in weeks 3–4 to support Tai Chi practice at home through behaviour change techniques (joint action & coping planning with carer) • Telephone contact by research team in weeks 2–18 to remind to attend classes if consecutively fail to attend 2 classes for unknown reason *Intervention led by carer:* • PWD to practise Tai Chi 20 mins per day • Daily self-monitoring & weekly instructor feedback: PWD to complete a daily diary of Tai Chi practised at home and hand to instructor at Tai Chi class weekly	• Joint action plan for practising Tai Chi at home • Joint coping plan for practising Tai Chi at home • Diaries of Tai Chi practised at home	• Increased participation in Tai Chi; physical activity designed to improve balance and prevent falls • Increased support to do Tai Chi via weekly instructor-led classes • Increased social support to practise Tai Chi through weekly contact with instructor and peers at the classes, and telephone reminders • Increased support to do Tai Chi at home via home booklet, homework sheets, action and coping planning, self-monitoring, instructor feedback, and alarm clock reminder	*Direct:* • Reduction in risk of falls via increased dynamic balance (postural stability) • Further reduction in risk of falls via: (a) increased functional balance (postural stability) (b) increased static balance (postural stability) (c) reduced fear of falls (d) delayed deterioration in global cognitive functioning (e) delayed deterioration in visual-spatial cognitive functioning • Reduction in risk of falls in the carer via: (a) increased dynamic balance (postural stability) (b) increased static balance (postural stability) *Indirect (*via *the above):* • Reduction in rate of falls • Increased quality of life • Increased quality of life in the carer • Reduced carer burden

**Table 2:** Tai Chi exercise prescription and instructional methods Evaluation Tool to transparently report the TACIT Tai Chi intervention [46]

Item	Description
Exercise prescription items	
Time	Each session will last 90 min; 45 min’ Tai Chi followed by up to 45 min’ informal discussion.
Length	20 week course designed for the trial.
Frequency	Weekly Tai Chi class.
Instructional method items	
Style	Old-frame Chen
Number of forms	8 warm-up patterns (Baduanjin) and 5 Tai Chi form patterns
Names of forms	8 warm-up patterns (Baduanjin): Note: Some patterns differ to the more common Baduanjin patterns found online and Chinese government sponsored Baduanjin, as the Elemental Tai Chi lineage is different. Patterns are refined as the course progresses. 1. Raising the Sky 2. Gathering the Heavens 3. Cow Looks at the Moon 4. Directing the Ocean (slightly adapted for older generation) 5. Shaolin Archer 6. Qi Gong Punching 7. Separating Heaven and Earth 8. Shaking the Earth 5 Tai Chi form patterns 1. Grand Ultimate Beginning 2. Immortal Pounds Mortar 3. Lazy to Roll Sleeves 4. Six Seals and Four Closes 5. Single Whip
Movement principles	The basic tenets of Tai Chi are emphasised throughout the course. Each class will emphasise good body posture, slow and controlled body movements, and correct joint positioning in regard to the knee (to never extend beyond the foot).
Breathing techniques	The Baduanjin will emphasise moving with the breath, with slow and controlled breathing during body movements. Breathing during the Tai Chi form will be encouraged to be natural with no specific breathing emphasised, because the addition of Buddhist breathing or Daoist reverse breathing would be too advanced for beginners. Each class will end with standing meditation.
Relaxation	The course itself is designed to elicit a mental state of calm without the requirement to explicitly instruct students to be calm. The meditation at the end of each class is also a relaxation exercise.
Progression	Progression of Tai Chi will be taught over the 20-week course. In particular, participants will be encouraged to start from their current level of physical ability and develop over the course (e.g. if cannot stand for the whole session to begin with, work toward being able to stand for the whole session). In addition, participants will gradually be taught the warm-up patterns and Tai Chi form patterns with repetition of all patterns every week. New warm-up and Tai Chi form patterns will be gradually introduced
Instructor credentials	Both instructors are experienced and have qualifications at senior instructor level for public Tai Chi classes.
Number of instructors	2
Unsupervised practice	Participants will be asked to practise Tai Chi at home 20 min per day (or if not possible then the equivalent across the week). Carers are to facilitate the person with dementia to practise Tai Chi at home. Home practice is encouraged by a 30-min home visit by the Tai Chi instructor and provision of coloured home exercise booklets and homework sheets for each week (see intervention section).
Additional information	The intervention is delivered each week using as its ethos 7 core principles: 1. Safety is paramount 2. Instruction is to be tailored to each participant’s capability 3. Participants are to do Tai Chi standing up (not seated) 4. Participants are to be challenged to progress in their physical ability (e.g. to hold positions for longer periods) 5. Classes will have a friendly and enjoyable environment 6. Weekly emphasis on the importance of home practice 7. Weekly invitation for participants to socialise at the end of each class with each other and talk with the instructor

##### Tai chi classes

Each session will last for 90 min, with 45 min instructor-led group Tai Chi followed by up to 45 min informal discussion. Dyads will be encouraged to participate in the informal discussions each week to foster mutual peer support, and provide opportunity for ongoing advice from the Tai Chi instructor in relation to the home-based exercise component. Up to 10 dyads may attend per class. Teaching is based on implicit learning techniques, drawing on the Positive Emotion-Motivated Tai Chi (PEM-TC) approach developed in the USA [25, 26]. Through repetition of movements and positive reinforcement, this approach capitalises on PWDs’ capacity to continue to learn motor tasks with the use of muscle or kinaesthetic memory, i.e., unconsciously through making behaviours automatic, despite impaired ability to explicitly recollect such memories [25].

##### Home-based tai chi exercises

Between the second and fourth class, the Tai Chi instructor will visit each dyad in their own home. Dyads will be given a pack containing a colourful home exercise booklet and weekly homework sheets, to serve as a reminder of what has been covered in the classes each week and to prompt practice of Tai Chi at home. The carers will be asked to facilitate the PWD to carry out Tai Chi for 20 min each day, at a convenient time and location. For safety reasons, dyads are asked not to practise Tai Chi at home until the Tai Chi instructor has made the home visit.

##### Behaviour change component

At the home visit the Tai Chi instructor will undertake a risk assessment of the environment where Tai Chi is being carried out, reiterate instructions on the performance of 20 min daily Tai Chi, and answers any queries. The Tai Chi instructor will re-affirm to dyads the benefits of doing Tai Chi and the central role of the home-based exercises to obtain these benefits, as recommended in NICE guidelines [27]. To enhance uptake and adherence to the home-based Tai Chi exercises, the instructor will then facilitate the dyad to make joint action and coping plans, which are based on self-regulation theory that has robust empirical support for increasing physical activity [28–32]. For action planning, dyads will decide together when and where they will do their Tai Chi exercises at home. For coping planning, the PWD and their carer will anticipate any personal barriers that may arise for them whilst carrying out the Tai Chi exercises at home and what they can plan to do to overcome them. The use of these behaviour change techniques has been recommended in NICE guidelines for physical activity promotion and behaviour change [27, 33, 34]. Other techniques recommended by these guidelines already embedded within the design of the intervention include self-monitoring (dyads will record their weekly completion of Tai Chi exercises), feedback on performance and adherence (from the instructor each week), and social support (from the instructor and peers in the class) [33].

In addition, the dyads will receive two forms of reminders that may help with their adherence to the TACIT Tai Chi intervention. First, they will be provided with a small branded alarm clock that they can set as a prompt to do their daily Tai Chi. Second, if dyads do not attend a Tai Chi class for two consecutive weeks for an unknown reason, at the next scheduled weekly telephone call with the PWD, the researcher will take the opportunity to remind the dyad of their Tai Chi class and encourage them to continue attending the class.

#### Assessment of treatment adherence

The Tai Chi instructor will keep a record of dyads’ weekly class attendance and a record of all home visits, along with a checklist to confirm all materials have been provided to each dyad. For home practice of Tai Chi, dyads will be asked to record their practice each week in a diary and return this to the instructor at the next class.

Qualitative data will be collected and reported separately from the main trial outcome paper. A researcher will observe 10% of the classes and make qualitative observations in relation to class-based adherence. At these observations a checklist will be used to confirm the Tai Chi instructors’ fidelity to the intervention protocol, and informal feedback from dyads and instructors will be sought at the end of each observation session and recorded using field notes. At around week 16 of the Tai Chi intervention, joint interviews will be conducted with a purposive sample of around 15 dyads in their homes and will focus on adherence to the intervention and in particular the home-based Tai Chi exercises.

#### Outcomes

The majority of measures will be taken at baseline and six months post-baseline (see Table 3). Weekly telephone calls with PWD for data collection will also serve as weekly contact to encourage retention. If one member of a dyad wishes to withdraw from the study, or is advised to withdraw by their general practitioner, they will be asked whether they are willing for data gathered up to the point of withdrawal to be retained for intention-to-treat analysis.

**Table 3:**
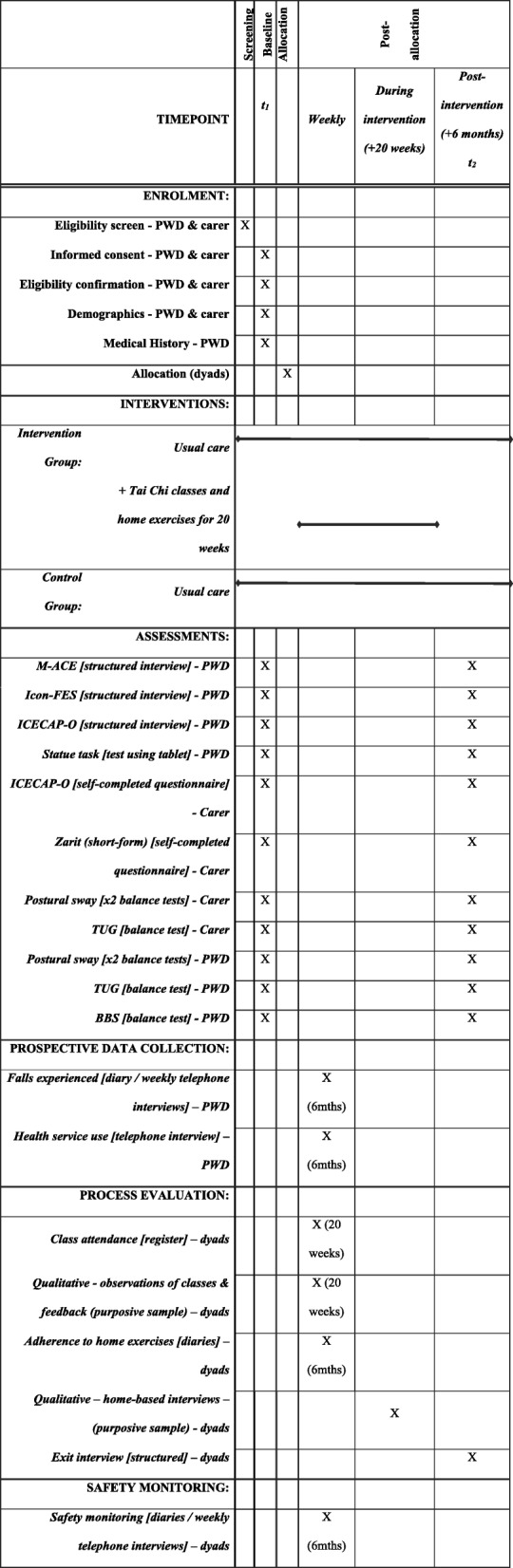
Study schedule for the RCT

Continuing participation of the other member of the dyad will be sought. Participants will not be replaced. The choice of key outcome measures was informed by a search conducted on The COMET database (Core Outcome Measures in Effectiveness Trials; www.comet-initiative.org). Both dynamic and static balance have been shown to be important modifiable risk factors for falling among community-dwelling older PWD [35], and can be measured with valid and reliable tools in this patient group [36, 37]. Descriptions of the primary and secondary outcome measures are presented in Table 4.

**Table 4:** Description of the outcome measures recorded at baseline and follow-up (six months post-baseline)

Item	What it measures	How it is measured	Unit of analysis	Justification
**Primary outcome**				
Difference in score from the person with dementia between the two arms at six months post-baseline:				
Timed Up and Go (TUG) test [64]	Dynamic balance	Continuous measure of time (in seconds) to complete the task. A cut-off point will not be used because there is no value that can be recommended from existing evidence [48, 49].	In addition to using a stopwatch, performance on the TUG will be measured using a Balance Sensor (THETAmetrix) that contains an accelerometer to digitally record biomechanical movement, and is a small, inexpensive device that is wireless and corrects for tilt dynamically. The data on the device will be downloaded immediately after each test and stored on the researcher’s laptop / tablet and labelled using the participant’s unique ID number.	The TUG is quick and simple to administer in the community [49] and has been recommended for screening for falls risk [50] and assessing gait and balance for preventing falls [51]. While no particular measure of dynamic balance has been recommended in the literature, systematic reviews have identified that the TUG has excellent reliability [42], a strong correlation with falls in retrospective studies [47], is more effective at ruling in falls (0.74 specificity) among individuals classified at high risk of falls [52], and is more suitable with older people who are relatively less healthy and have lower functioning [48]. Devices such as the balance sensor have been shown to produce reliable and valid data for the TUG and its subcomponents [53, 54].
**Secondary outcomes**				
Difference in score between the two arms at six months post-baseline on the following:				
Person with dementia - ×2 balance tests				
Berg Balance Scale (BBS) [55]	The BBS is an objective measure designed to assess functional balance and fall risk in adult populations [55]. The BBS takes an overall assessment of an individual’s balance; “underlying motor systems, static stability, dynamic stability, functional stability limits, anticipatory postural control, and sensory integration” p. 13 [56].	This is a 14 item scale with a 5-point response for each item (0–4), with the sum score used (minimum to maximum possible scores of 0–56, with 0–20 high fall risk, 21–40 medium fall risk, and 41–56 low fall risk).	Total score will be analysed (potential range 0–56) and will be assumed to be interval scaled.	It has been recommended in a recent consensus as one of two core outcome sets for measuring standing balance in adult populations [56]. This consensus reported that this scale would be more useful among those with limited functioning (it is prone to ceiling effects among the generally healthy population) [56]. We chose the BBS for this study based on its likely ease of use among people with dementia, existing published evidence of its suitability for use with people with dementia [36, 57], and its feasibility for use in people’s homes.
Postural sway while standing on the floor and on a foam mat [35]	Static balance under usual and challenging conditions	In both instances, a continuous value will be measured as total (antero-posterior + medio-lateral) normalised path length of the acceleration sway trace of the pelvis during the task. This will be recorded using a Balance Sensor (THETAmetrix), mounted over the upper sacrum (s2 spinous process) to digitally record body sway.	The unit of measurement will be in milli-g/second (mg/s).	The sensor is quick to use (2mins per test) and been shown to be as reliable as laboratory forceplates [58, 59].
Person with dementia – × 4 structured interview scales				
Iconographical Falls Efficacy Scale (Icon-FES, short form) [60]	Fear of falling	This is a 10-item scale of fear of falling with a 4-point response for each question (1–4).	Sum score (minimum to maximum possible scores of 10–40, higher scores indicating greater fear). It will be assumed that this is interval scaled data (scale of 10–40).	The Icon-FES is better at identifying people at higher risk of falls compared with the Falls Efficacy Scale-International and does not produce a floor effect [60].
Mini-Addenbrooke’s Cognitive Examination (M-ACE) [22]	Brief measure of global cognitive functioning	Five items: attention (assesses orientation, scored 0–4), memory (scored 0–7), fluency (assesses language, scored 0–7), visuospatial function (scored 0–5), and memory (assesses recall, scored 0–7), with a total score of 0–30.	The sum score is used, with values on an interval scale of 0–30 with higher scores indicating greater cognitive function.	The M-ACE is more sensitive than the Mini Mental State Examination and is less likely to have ceiling effects, which makes it particularly useful with people with mild cognitive impairment [22].
Statue task (Reed & Spiers: Development of a spatial judgement task for use in Alzheimer’s disease: The effect of permanency in spatial environments with age, unpublished)	Brief measure of visual-spatial cognitive functioning that uses a tablet to administer the task (Reed & Spiers: Development of a spatial judgement task for use in Alzheimer’s disease: The effect of permanency in spatial environments with age, unpublished).	Presents participants with a series of visual scenes. The participant is asked to look at scenes with three statues and a stool, and to answer a series of questions that assesses their ability to perceive the objects in three-dimensional space and their relationships to each other. The computer automatically records the time taken to complete the task and number of errors made.	A continuous measure is used for time taken to complete (in seconds) and a discrete measure for the number of errors made (frequency count).	This is a measure of specific cognitive functioning from the hippocampus, which is therefore a more sensitive measure to change than a global assessment of cognitive functioning.
ICEpop CAPability measure for Older people (ICECAP-O) [61]	Quality of life	5 item scale with a 4-point response for each (1–4)	Sum score used (minimum to maximum possible scores of 5–20 with higher scores indicating greater capability). It will be assumed the measure is interval scaled.	This measure is from the perspective of capability to be independent, which is associated with fall risk, general balance and mobility, and sensitive to cognitive status [62]. It is also a measure recommended in guidelines on economic evaluation of fall prevention interventions [63], with results that can be compared with other economic evaluations that used the ICECAP-O.
Carer – ×2 balance tests				
Timed Up and Go (TUG) test [64]	As above	As above	As above	As above
Postural sway while standing on the floor and on a foam mat [35]	As above	As above	As above	As above
Carer – × 2 structured interview scales				
ICEpop CAPability measure for Older people (ICECAP-O) [61]	As above	As above	As above	As above
Zarit Burden Interview (short-form) [65]	Carer burden	12-item scale with a 5-point response for each (0–4)	Sum score used (minimum to maximum possible scores of 0–48 with higher scores indicating greater burden). An assumption will be made that the data are interval scaled.	The most commonly used tool for carer burden [66], and is shorter but just as reliable and valid as the full-length version [65, 66].

Falls measurements will be collected prospectively from baseline until six months post-baseline. These include the number of falls by PWD (count data), the proportion of PWD who have fallen (binary outcome measure), and number of injurious falls (count data). The collection of falls data is in accordance with recommendations made by the Prevention of Falls Network Europe (ProFaNE) [38]. In our trial, the definition of a fall is, “an unexpected event in which the participants come to rest on the ground, floor or lower level” [38]. Falls will be recorded by dyads daily using prospective monthly calendars and returned on a monthly basis by post for data entry. Where falls are reported by dyads or calendars are not returned, a researcher will conduct a telephone interview to collect further information about the fall/clarify missing data. Telephone calls will be conducted weekly with the PWD as recommended for falls data collection with PWD [39]. In addition, to ascertain the accuracy of different recall periods, telephone calls about fall incidents by the PWD will be made monthly with the PWD and every 3 months with the carer. Fall injury will be recorded by telephone interview when recording falls as described above, using an existing set of definitions for severity of injury ([11, 40], p.).

Within the intervention group only, data will be collected half-way through the intervention on a 5-item questionnaire to ask about the importance of features of the intervention such as enjoyment of the classes and confidence in being able to continue to do the home practice. At the end of the final home visit, all dyads will be asked to complete a brief structured exit interview that asks about their hypothetical willingness to pay for the Tai Chi intervention should it be offered in the future as part of routine NHS care. The researcher will also ask participants if there have been any significant changes during the trial to their medical status (e.g. medication usage or elective surgery), which may influence performance on the primary outcome.

#### Sample size

A formal sample size calculation was undertaken based on detecting a difference in mean timed up and go (TUG) test scores between the intervention and control arms of the study. We found no studies looking at minimum clinically important difference, but did find two studies that had estimated smallest detectable change (i.e. the smallest change that we can be reasonably sure is not measurement error). The two values were 4.09 [41] and 5.88 [37], and we used a conservative value of 4. To find an estimate of standard deviation (SD) for TUG, we found several relevant papers but the values were sensitive to the mix of age and severity of dementia, with one study that seemed to most closely match our population and be based on a reasonable sample size (*n* = 58) [37]. SDs from two different time points were presented (9.74 and 9.01) and we used the average of these (9.38) [37]. In our analysis we will enter baseline TUG as a covariate and assume a correlation of 0.7; test-retest reliability of TUG is excellent [37, 42] but we acknowledge the duration between time points in our study will be longer than prior studies and so we have used a more modest correlation.

Therefore, using a mean difference in TUG of 4, an SD of 9.38, a correlation of 0.7 and a 2-sided 5% significance level, the study will have 90% power when the sample size is 120 (60 per group). Allowing for up to 20% withdrawal / non-completion of outcome measures, we will recruit 150 dyads into the trial (75 per group). These calculations were conducted using nQuery Advisor 7.0. The intervention partly takes place in classes, and there is a possibility of clustering effects. However for the purposes of the sample size calculation we have assumed that these will be negligible, because (a) much of the intervention occurs outside the class environment, and (b) interaction between PWD is not a specific purpose of the intervention’s effect on the primary outcome (only to enhance adherence to the intervention). Clustering effects will be considered in the statistical analysis.

#### Recruitment

Recruitment of participants will utilise multiple pathways, including:

- Contacting PWD identified from searches on NHS research, NHS clinic, and voluntary sector databases.
- Opportunistic recruitment of patients using NHS memory assessment service clinics, NHS older people mental health services, NHS outpatient clinics, voluntary sector memory advisors, and GPs.
- Recruitment via the Join Dementia Research website, endorsed by the Health Research Authority, to facilitate patient recruitment into dementia studies (http://jdr-delivery.nihr.ac.uk/).
- Recruitment via potential participants’ direct responses to study promotion including leaflets/posters in dementia cafés, support groups, general practices, chemists, pharmacies, day-care centres, newspapers, radio, social media, and informal newsletters.

All potential participants will be asked if they are willing for their contact details to be provided to a member of the TACIT research team.

A member of the TACIT research team will contact potential participants to arrange an initial visit, typically at the potential participant’s home. The researcher will wait a minimum of 48 h before contacting potential participants to ensure they have had sufficient time to consider the information in the Participant Information Sheet. Both PWD and carer must be present at the initial visit and both must provide written informed consent. Researchers will use process consent with PWD, which is established in the evidence base [43–45], in that consent will not be assumed for the duration of the visit or for the project after provision of initial informed consent. Informed consent will be re-confirmed verbally throughout the project.

After consent has been obtained, the researcher will administer the M-ACE to the PWD to confirm that they meet the entry criterion in relation to dementia severity. As version A is used in routine care, version B will be used at baseline and version C at follow-up (to reduce potential bias due to practice effects). Participants recruited from outside NHS / GP services will be required to sign a consent form to enable the research team to verify the PWD’s dementia diagnosis (type and when diagnosed) from their memory assessment service or provide written evidence of their diagnosis.

### Assignment of interventions

In this trial we will aim to achieve equal balance of recruitment of participants between treatment conditions according to the following key prognostic variables: dementia type (due to eligibility criteria, this will be relatively homogenous in this study), age, fall history, and dementia severity. These are important variables because impairment in postural stability will be elevated among those who are older, have fallen in the past 12 months, and present with more severe dementia symptoms [19, 20]. Given that these key prognostic variables are positively correlated [19, 20], only one variable - fall history at baseline: has / has not fallen within the previous 12 months – was selected for a parsimonious and achievable strategy for balancing prognostic factors. The method of allocation needed to accommodate a trial design that allowed for variability in Tai Chi class size according to a dynamic recruitment process. Therefore, to ensure balance within each of the three treatment sites (and across the entire trial), minimisation will be used within each site to achieve a balance in fall history.

For each of the 11–15 classes, once a minimum of 4 dyads in total have been recruited, the dyads will be randomised to the intervention or control group. Random allocation will be achieved using a centralised automatic web-based randomisation system designed and maintained by the UKCRC-registered Peninsula Clinical Trials Unit (CTU). Once a minimum batch of 4 dyads has been recruited within a treatment site, the first dyad will be allocated using simple randomisation and the remaining dyads allocated using minimisation. Afterward, any further dyads recruited into the study before the fifth class has taken place will be randomised individually by minimisation and join the class if so allocated.

#### Outcome assessor blinding

Baseline measures will be recorded at the PWD’s home before randomisation is performed. After the home visit, dyads will be informed in writing of their treatment arm allocation by staff at the CTU. Given the nature of the intervention, it will not be possible to keep PWD, carers or Tai Chi instructors blind to treatment arm allocation. The researcher who collects baseline data will be kept blind to treatment arm and, in particular, when they conduct the six-month post-baseline follow-up home visit. Participants will be asked at the time of arranging the follow-up visit and at the beginning of the follow-up home visit not to disclose their treatment condition, and to conceal any evidence of participating in class or home-based Tai Chi. After data collection is completed for each follow-up visit, the researcher will make a note of their guess of the treatment arm for the dyad and if dyads accidentally disclose their treatment condition.

#### Blinding of data analysis and interpretation

Copies of the completed case report forms (CRFs), labelled with a unique trial number for each dyad member, will be sent to the CTU for double data entry into a web-based trial database designed and maintained by the CTU. The data will be exported for the statistician with treatment condition unblinded since information on class cohort is required for the analysis. Once the analysis is complete, the results will be revealed to the rest of the research team with trial arm identity concealed. After the results have been discussed and interpreted, the results will be unblinded for the research team to complete their interpretation and to begin dissemination of the findings.

### Data collection, management, and analysis

The CTU will seek clarification on any data queries with the researcher that will prompt the researcher to seek clarification from the PWD at the next scheduled telephone call. All data will be stored according to the UK Data Protection Act and the General Data Protection Regulation. Anonymised and identifiable study data will be stored separately to prevent the identification of participants from research records, in locked filing cabinets within a locked office. All digital records will also be securely stored on backed up university servers with restricted access. Data held on BU laptops will also be protected through encryption and network authentication and transferred to the secure trial folder as soon as possible. Data will be double-entered by CTU staff on to a password-protected SQL Server database and encrypted using Secure Sockets Layer.

#### Statistical methods

A detailed statistical analysis plan will be finalised separately before follow-up data collection is complete. Participants will be analysed in the group they are randomised to, and (with the consent of participants) we will attempt to collect complete data on everyone and use those data in the analyses. Descriptive statistics will be presented for all baseline outcome data for each trial arm separately. Descriptive statistics will be presented on the following for the PWD and their carer: gender, age, relationship status, current living arrangements, education level, ethnicity, previous experience of Tai Chi and treatment site. Additional descriptive statistics will be presented in relation to PWD: type of dementia, time since diagnosis, other long term conditions, existing injuries, use of a walking aid, number of medications currently taken, falls history (in past 12 months and past month), and current level of physical activity (moderate and vigorous).

Mean TUG scores at 6 months, the primary outcome, will be compared between the two trial arms using a mixed (multi-level) model approach to take into account clustering resulting from the group-based nature of the Tai Chi intervention. Baseline TUG, treatment site, and falls history in the past 12 months (Y/N) will also be included in the model. We will assume that the missing data mechanism is “Missing at Random” (MAR). No imputation methods will be used for the main analysis but will be explored in additional analyses. The method of analysis will be similar for other secondary outcomes for the PWD and carer. In addition, falls count data and the proportion of participants who fell will be analysed using negative binomial and logistic models respectively, taking into account falls history and treatment site. We will also conduct a per protocol analysis that will exclude participants from the Tai Chi group if they received fewer than 50 h (the intended minimum dose of the intervention).

One subgroup analysis is pre-planned for the primary outcome variable (TUG). Fall history at baseline (had a fall or not in the past 12 months), which is also the variable used for minimization in addition to treatment condition, will be entered as an interaction term in a statistical model (intervention effect x effect modifier). Departures from the proposed analysis, for example due to poor recruitment, will be explained and justified in the main trial paper. Feasibility objectives will be addressed by estimating recruitment and attrition rates (with 95% confidence intervals) and falls data parameters (falls rate, proportion of fallers, and completeness of falls data) for a future sample size calculation. In addition, we will compare the frequency of falls reported by each of the individual methods of data collection and their combination to determine if the increased resource in collecting falls data weekly provides more accurate data.

Descriptive data from the structured exit interviews will be presented on hypothetical willingness to pay for the intervention and perceived changes to health (PWD and carer). Data on number of Tai Chi classes attended and adherence to home practice will be presented. To assess adherence to the intervention in the Tai Chi arm we will calculate: adherence to classes (i.e. x attended out of y possible) and adherence to home exercise (i.e. x minutes exercise out of y recommended). The following data will not be reported in the main trial outcome paper but will be included in further secondary analyses in relation to visual spatial cognitive functioning (statue task); comparison of the different methods of data collection on incidence of falls; qualitative data; and uptake and adherence to the intervention (whether the PWD or carer had done Tai Chi before, mean (SD) intention and perceived behavioural control to do Tai Chi, and intervention group responses to the questionnaire delivered half way through the intervention).

Given the short-term (6-month) follow-up, low-risk intervention being tested, and no planned interim analyses, there will be no data monitoring committee for the trial. The trial will only be stopped early if the intervention is considered to be directly causing undue harm to patients as independently considered by the trial steering committee and/or NHS Research Ethics Committee.

#### Health economic analysis

We will assess the feasibility of collecting the data required for a health economic analysis. This will include detailed descriptive statistics on completion of the health service use telephone interviews, which will include social care costs and costs incurred by dyads. Health service use costs will include both those from falls (both intervention arms) and in relation to injury while carrying out Tai Chi. The total cost of providing the intervention to each patient will be estimated from weekly records collected from the Tai Chi instructor and in relation to: (a) the average cost of hire of the building for the classes (taken from the costs incurred in this study), and (b) the instructor’s time spent delivering the intervention as a function of hourly rate. Health service use on falls will be calculated in relation to presentation to the GP, out of hours, or ED. We will also explore cost effectiveness results for PWD and their carers between arms in terms of incremental cost per change in quality of life. All health economic analyses will be presented descriptively in terms of completion (missing data) and outcomes (e.g. mean and SD in relation to baseline and follow-up), as the study has not been powered to conduct a full health economic analysis, which is reserved for the subsequent definitive trial.

#### Safety monitoring

The monitoring of participants’ safety will be achieved primarily through weekly telephone contacts between unblinded researchers and dyads and dyads’ weekly record keeping. Participation in the trial is considered to be of relatively low risk and participants are not expected to be harmed as a result of taking part. As such, the recording and reporting of adverse events will be limited to adverse events related to the Tai Chi intervention and balance test assessments. No treatment will be provided to participants in addition to NHS usual care.

#### Responsibilities of the trial steering committee (TSC)

The TSC will oversee the conduct and safety of the trial, ensuring that milestones are achieved and general scientific probity is maintained. The TSC will meet by teleconference on a bi-annual basis. The TSC will review progress against key milestones and reports of aggregate data (blinded) with a particular focus on adverse events. The TSC will be independently chaired and include independent members from the trial that represent expertise in statistics, clinical care of older people, relevant research knowledge and experience, and representation of older people with dementia and their informal carers.

### Ethics and dissemination

#### Access to data

Anonymised electronic records and paper copies of records will be open to inspection and monitoring from a recognised representative from either the Sponsor, Bournemouth University, the CTU, or the funder (National Institute for Health Research). The trial management group will have access to the full dataset. Other interested parties may make a formal request to access the electronic dataset, which will be approved / declined by the CI in accordance with the Data Management Plan that will detail management of access, sharing, and preservation of the data. Any use of the electronic data set must comply with the dissemination policy (see below) and be requested via Bournemouth University Library (bordar@bournemouth.ac.uk) who will collaborate with the CI with regards to access. Non-digital data supporting this study will be stored by the corresponding author at Bournemouth University. Only electronic data will be shared with bona fide researchers intending to use the data for non-commercial research purposes, after an embargo period of approximately 24 months. Access to the following will be restricted to researchers who sign a confidentiality agreement and confirm their intention to use the data is for secondary data analysis for non-commercial research purposes using a Creative Commons licence: statistical analysis plan; where applicable, statistical code (for final analysis of primary outcome measure); and anonymised participant-level dataset and data documentation.

#### Dissemination policy

We intend to notify participants involved in the trial of the study findings through our annual public engagement events and a newsletter on study completion. We will disseminate to researchers and healthcare professionals through conference presentations and scientific journal publications, and the general public through our webpage for the trial (www.bournemouth.ac.uk/tai-chi). In all dissemination, no professional writers will be used. To establish authorship eligibility, we will use the uniform requirements for manuscripts submitted to biomedical journals established by the International Committee of Medical Journal Editors (ICMJE; www.icmje.org).

## Discussion

### Public and patient involvement (PPI)

PPI informed the development of the grant application for the trial. In particular, we held a small group discussion with two PWD and four carers (two carers were spouses and two were daughters of men with dementia). The idea of doing a Tai Chi intervention was presented to the group, with an explanation of Tai Chi provided by a qualified instructor. The group found the idea of a Tai Chi intervention appealing, and influenced the application in four ways: The inclusion of informal carers in dyads beyond partners / spouses; the provision of weekly classes for the whole 20-week course; the importance of ease of transport to each venue; and asking dyads about hypothetical willingness to pay for the intervention at the exit interview. Since the project began, a PPI advisory group of ten people (4 PWD, 5 spouses of PWD, and a daughter of a PWD) have inputted into the development of this trial protocol and continue to provide PPI input throughout the trial at regular intervals.

### Progress to date

At the time of submission, the trial is open to recruitment and the first dyad was recruited on June 7th, 2017. The title of the trial for the public is, “The TACIT Trial: TAi ChI for people with demenTia”. YB-M is the contact for all public queries.

## Abbreviations

BBS: Berg Balance Scale
CI: Chief Investigator
ED: Emergency Department
ICECAP-O: ICEpop CAPability measure for Older people
Icon-FES: Iconographical Falls Efficacy Scale
M-ACE: Mini-Addenbrooke’s Cognitive Examination
NHS: National Health Service
PPI: Public and Patient Involvement
PWD: People with dementia
RCT: Randomised Control Trial
SD: Standard deviation
TSC: Trial Steering Committee
TUG: Timed Up and Go test
